# The Guanidine Pseudoalkaloids 10-Methoxy-Leonurine and Leonurine Act as Competitive Inhibitors of Tyrosinase

**DOI:** 10.3390/biom10020174

**Published:** 2020-01-23

**Authors:** Jang Hoon Kim, Hyun Hee Leem, Ga Young Lee

**Affiliations:** 1 Research Institute for Basic Sciences, JeJu National University, Jeju 63243, Korea; 2 National Development Institute of Korean Medicine, Gyeongsan 38573, Korea; npb0391@nikom.or.kr; 3 Hephzibah Korea Lnc, Techno 10-ro, Yuseong-gu, Daejeon 34036, Korea

**Keywords:** *Leonurus japonicas*, guanidine pseudoalkaloid, tyrosinase, competitive inhibitor, lead compound

## Abstract

Tyrosinase plays a key role in the production of melanin. A variety of industrial fields have shown interest in the development of tyrosinase inhibitors from plants. In this study, compounds **1**–**5** derived from *Leonurus japonicas* were evaluated to determine their ability to inhibit tyrosinase. Of these, 10-methoxy-leonurine (**1**) and leonurine (**2**) exhibited IC_50_ values of 7.4 ± 0.4 and 12.4 ± 0.8 μM, respectively, and acted as competitive inhibitors of tyrosinase, with *K*i values in the micromolar range. In silico modeling revealed a guanidine group located in the inner cavity and a benzene ring docked within the active site of these compounds. These guanidine pseudoalkaloids show potential not only as tyrosinase inhibitors but also as lead compounds in new scaffolds for the development of novel inhibitors.

## 1. Introduction

Tyrosinase (EC 1.14.18.1), found in bacteria, fungi, plants, and mammals [1], is a copper-containing metalloenzyme with various catalytic functions, such as hydroxylation of monophenols and oxidation of diphenols, which are important for the production of melanin pigment [2]. Excessive accumulation of melanin is responsible not only for pigment disorders in human skin but also enzymatic browning of fresh-cut fruits and vegetables [3]. It is also important for cuticle formation in insects [4]. Inhibiting the production of melanin precursors to prevent the undesirable effects of melanin is considered an important strategy [4]. As a result, tyrosinase is regarded as a target enzyme for the development of whitening agents, food preservatives, and agricultural insecticides [5]. In addition, tyrosinase inhibitors have been also proposed as neuroprotective agents [6]. Recently, secondary metabolites isolated from natural products, such as aloe-emodin [4], broussoflavonol J, broussoflavonol H [7], and calycosin [8], have been evaluated as tyrosinase inhibitors because of their low toxicities.

*Leonurus japonicas* is a perennial herb of the Labiatae family widely distributed in Asia [9]. This plant, which is referred to as “Yi Mu Cao” in traditional Chinese medicine, has been used to treat menoxenia, dysmenorrhea, amenorrhea, and ulcerations [10]. Phytochemical investigation of *L. japonicas* has led to the isolation of a variety of compounds from this plant by column chromatography, including ladbane diterpenes [11], phenylethanoid glycosides [12], steroids [13], alkaloids [14], and flavonoid glycosides [14]. Among the isolated compounds isolated, studies showed that leojaponin inhibited melanin production in B16F10 melanoma cells [15], and that phenylethanoid glycosides exerted hepatoprotective activity similar to that of bicyclol in HL-7702 cells [12].

The objective of this study was to evaluate the inhibitory activities of compounds from *L. japonicas* on tyrosinase. The pseudoalkaloids 10-methoxy-leonurine and leonurine, at micromolar concentrations, were revealed to be potential inhibitors of tyrosinase. These compounds disrupt the catalytic reactions of tyrosinase by binding to its active site. Molecular docking simulations showed that these compounds fit into the active site of the enzyme. Thus, pseudoalkaloids may be lead candidates for the development of tyrosinase inhibitors from *L. japonicas.*

## 2. Materials and Methods

### 2.1. General Experimental Procedures

ESI-MS spectra were recorded on a microTOF-QTM II (Bruker, Billerica, MA., USA). NMR experiments were conducted on an ECA500 (JEOL, Tokyo, Japan), with the chemical shift referenced to the residual solvent signals. TLC analysis was performed on Kieselgel 60 F254 (Merck, Darmstadt, Hesse, Germany) plates (silica gel, 0.25 mm layer thickness), single compounds were visualized by dipping plates into 10% (*v*/*v*) H_2_SO_4_ reagent (Aldrich), and then dried air heat treated at 300 °C for 30 s. Silica gel (Merck 60A, 70 to 230 or 230 to 400 mesh ASTM), Sephadex LH-20 (Amersham Pharmacia Biotech, Darmstadt, Hesse, Germany), and reversed-phase silica gel (ODS-A 12 nm S-150, S-75 μm; YMC Co., Kyoto, Kansai, Japan) were used to perform open column chromatography. Tyrosinase (T3824) and l-tyrosine (T3754) were purchased from Sigma-Aldrich (St. Louis, MO, USA).

### 2.2. Extraction and Isolation

*L. japonicus* (5 kg) was extracted with 80% methanol at 27 °C. The concentrated methanol extract (0.8 kg) was suspended in distilled water and successively partitioned with n-hexane, ethyl acetate, and aqueous layers. The ethyl acetate fraction (73.1 g) was subjected to silica gel column chromatography with a gradient of *n*-hexane-acetone gradient (from 10:1 → 1:100) to yield 20 fractions. E 18 fraction was purified with C-18 column chromatography with methanol gradient (from 30% → 50%) to give compound **3** (72.1 mg). Compound **4** (21 mg) was purified by C-18 column chromatography with 50% methanol isocratic system from E 19 fraction. E 20 Fraction was chromatographied by C-18 column chromatography with 50% methanol to gain six fractions (E201–206), and then E203 fraction was purified with Sephadex LH-20 column chromatography by using 40% methanol to obtain compounds **1** (15.1 mg), **2** (8.5 mg), and **5** (51.9 mg). The spectrum analysis is in the Supplementary Materials.

### 2.3. Enzymatic Assay

The enzyme assay was performed with spectrophotometer as described previously with a modified method [16]. Briefly, 130 μL of tyrosinase in 0.05 mM phosphate buffer (pH 6.8) and 20 μL of the inhibitors dissolved in MeOH were added into a 96-well plate, and then mixed with 50 μL of l-tyrosine at 37 °C. The mixture was recorded at wavelength of 475 nm for 20 min.

The inhibitory ratio was calculated according to the following equation:

Inhibitory activity rate (%) = [(ΔC/ΔS)/ΔC] × 100(1)

where ΔC and ΔS represent the intensity of control and inhibitor after 20 min, respectively.

y = y_0_ + [(a × x)/(b + x)] (2)

where y_0_ is the minimum value on the y-axis, a denotes the difference between maximum and minimum values, and b refers to the x value at 50%.

1/*v*_0_= (*K*m/*V*max)(1 + [I]/*K*_i_)(1/[S]) + 1/*V*max (3)

where *v*_0_ represents the initial velocity, *V*max is the maximum rate at saturation of the substrate concentration, and *K*m is the substrate concentration [S] at which the reaction rate is half of *V*max.

1/*v*_0_ = (*K*m/*V*max[S]*K*_i_)[I]+1/*V*max(1+*K*m/[S]) (4)

where *K*_i_ is the inhibition constant of the inhibitor binding to the enzyme.

### 2.4. Docking Simulation

This study was performed as previously described [5]. The three-dimensional structure of tyrosinase was achieved from the file coded in 2Y9X from RCSB for docking simulation, and then water and substrate molecules in pdb file were deleted. The structure of inhibitors was constructed by the minimization with MM2. Pseudoalkaloids (**1** and **2**) were docked with the grid containing the activity site (number of points: *X*: 80, *Y*: 80, *Z*: 80) by Autodock 4.2. Docking of inhibitor into active site of protein was performed by using the Lamarckian Genetic Algorithm (runs: the maximum number of evals was set as long). The simulation results were prepared using Chimera and LigPlot.

### 2.5. Data Analysis

Data were expressed as the means ± standard deviation (*n* = 3). All values were analyzed using SigmaPlot (Systat Software Inc., San Jose, CA, USA) to determine treatment variations.

## 3. Results

### 3.1. Identification and Determination of the Tyrosinase Inhibitory Activity of Compounds

**Figure 1:**
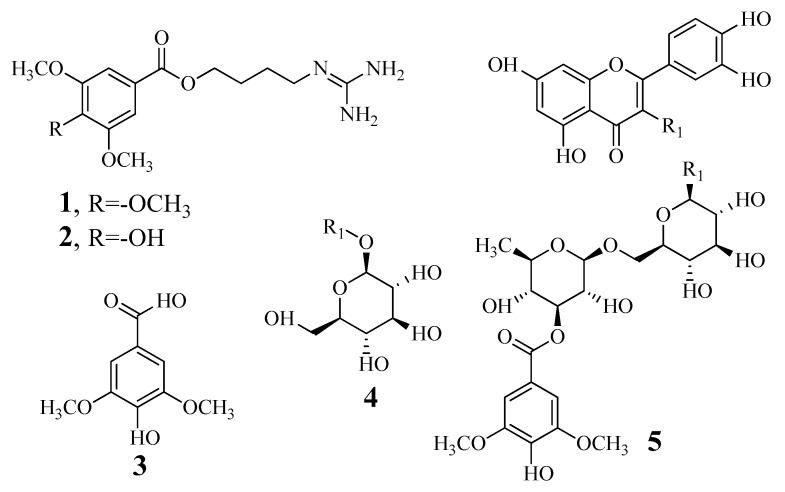
The structure of components **1**–**5** from *Leonurus japonicas*.

Compounds **1**–**5** were isolated from *L*. *japonicas* by column chromatography. The structures were elucidated by nuclear magnetic resonance spectroscopy and compared with previously reported mass spectra. The compounds were determined to be 10-methoxy-leonurine (**1**) [17], leonurine (**2**) [17], syringic acid (**3**) [17], isoquercitrin (**4**) [18], and leonurusoide E (**5**) [18] (Figure 1).

**Figure 2:**
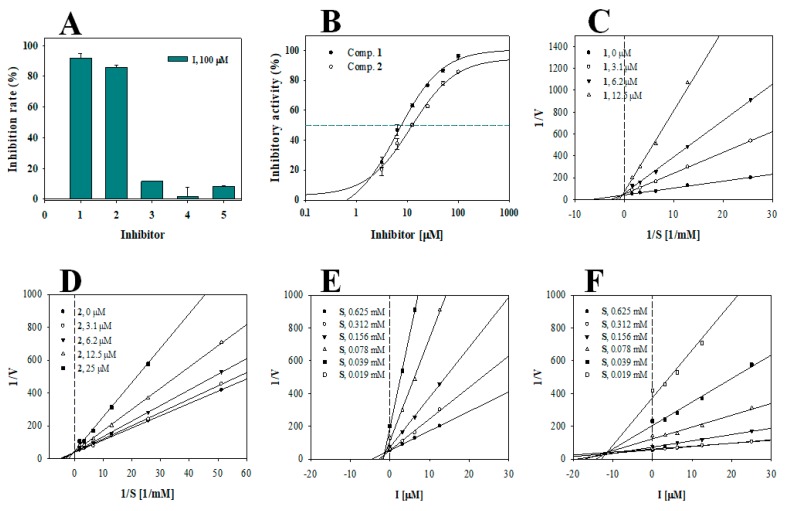
The inhibitory activities of compounds **1**–**5** at 100 μM concentration toward tyrosinase (**A**). IC_50_ values (**B**), Lineweaver-Burk plots (**C**,**D**), Dixon plots (**E**,**F**) of two inhibitors **1** and **2**, respectively.

The tyrosinase inhibitory activities of compounds **1**–**5** were evaluated. To screen the isolated compounds, their tyrosinase inhibitory rates were evaluated at a compound concentration of 100 μM and were then calculated using Equation (1). Compounds **1** and **2** showed greater than 80% inhibitory activity, and IC_50_ values were determined (Figure 2A, Table 1). The dose-dependent inhibitory rates of 10-methoxy-leonurine (**1**) and leonurine (**2**) at concentrations ranging from 3.1 to 100 μM were evaluated. Six inhibitory rates were input into Equation (2), and the IC_50_ of the inhibitor was defined when Equation (2) was equal to 50. The IC_50_ values of 10-methoxy-leonurine (**1**) and leonurine (**2**) were determined to be 7.4 ± 0.4 and 12.4 ± 0.8 μM, respectively (Figure 2B, Table 1).

### 3.2. Enzyme Kinetics

To analyze the binding of the inhibitors to tyrosinase, 10-methoxy-leonurine (**1**) and leonurine (**2**) were subjected to enzyme kinetic analyses. The rate of substrate to product conversion via interactions of the substrate and inhibitor with tyrosinase is dependent on the concentrations of the substrate (0.04–0.62 mM) and inhibitor. The initial velocity (*v*_0_) was induced within 15% of all conversion rates. The results are presented as a Lineweaver-Burk plot of 1/*v*_0_ versus 1/[S] to determine the binding mechanism. 10-methoxy-leonurine (**1**) and leonurine (**2**) made a family line induced by Equation (3). As shown in Figure 2C,D, inhibitors **1** and **2** had different −1/*K*_m_ and 1/*V*_max_ values (Figure 2C,D, Table 1), suggesting that these compounds are competitive inhibitors. Furthermore, a Dixon plot using Equation (4) revealed that the inhibition constants (*K*_i_) for inhibitors (**1**) and (**2**) were 1.6 ± 0.7 and 11.4 ± 1.1 μM, respectively (Figure 2E,F, Table 1).

### 3.3. Molecular Docking

**Figure 3:**
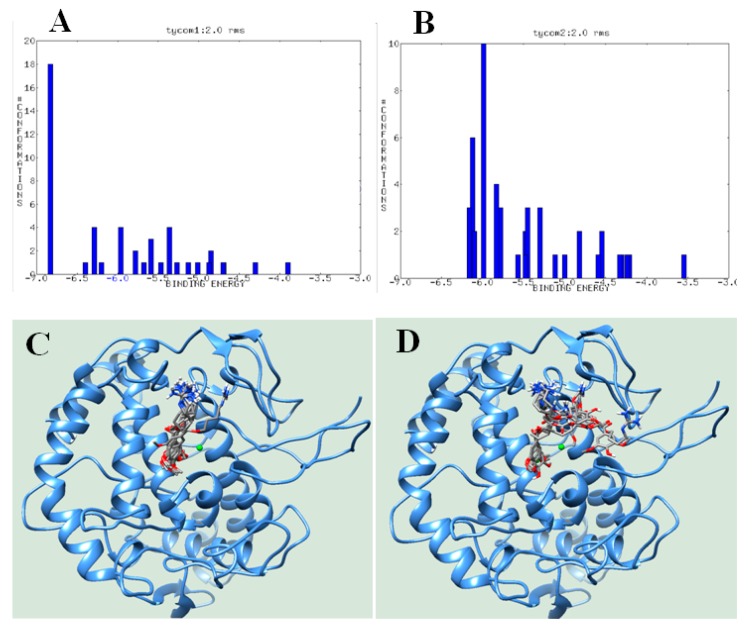

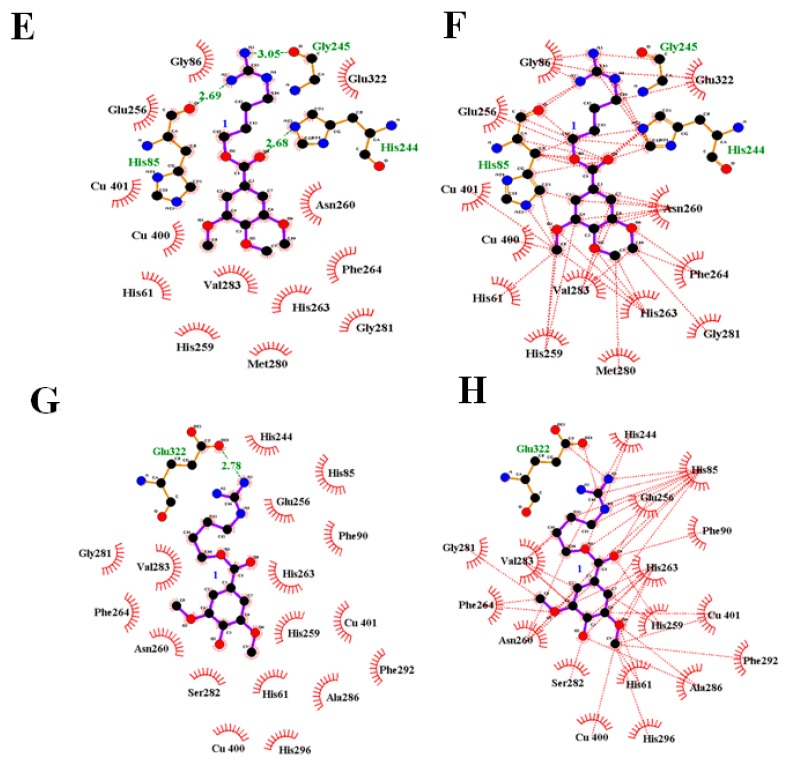
Cluster analysis of Autodock scores of inhibitors **1** and **2** into tyrosinase (**A**,**B**). The overlapped docking pose of the top 10 ranks (**C**,**D**). Hydrogen bonds (**1**: (**E**,**G**)) and hydrophobic interaction (**2**: (**F**,**H**)) at the best pose of the two docked into the catalytic site.

Our enzyme kinetic analysis suggested that compounds (**1**) and (**2**) dock within the active site of tyrosinase. We performed molecular docking simulations using the Autodock 4.2 program to determine the binding site between ligand and receptor. They were attempted in the simulation into he grid containing active site 25,000,000 times. We then extracted and analyzed the top 50 Autodock scores (Figure 3A,B, Table 2). 10-methoxy-leonurine (**1**) had a cluster at approximately −6.8 kcal/mol, whereas leonurine (**2**) had two clusters at approximately −6.2 and −6.1 kcal/mol. The top 10 ranks of the two inhibitors (**1** and **2**) were also analyzed (Figure 3C,D, Table 2).

The lowest Autodock score (−6.83 kcal/mol) for 10-methoxy-leonurine (**1**) revealed three hydrogen bonds with His85 (2.69 Å), His244 (2.68 Å), and Gly245 (3.05 Å) and hydrophobic interactions with 16 amino acids. In the simulation, leonurine (**2**) (Autodock score of −6.16 kcal/mol) maintained a distance of 2.78 Å from Glu322 and interacted with 10 residues (Figure 3E–H, Table 2).

## 4. Discussion

The search for new tyrosinase inhibitors is of interest in medicinal, cosmetic, agricultural, and food industries [6,19]. Tyrosinase catalyzes the reactions that produce dihydroxyphenylalanine and dopaquinone in the initial steps of melanogenesis [20]. Diterpenes and methanol extracts from *L*. *japonicus* were shown to stimulate melanogenesis in B16F10 cells [15]. The guanidine pseudoalkaloid leonurine (**2**) was first identified in 1930 as an important compound of *Leonurus sibiricus* [21]; leonurine decreases the interleukin-1β-induced expression of cyclooxygenase-2 and inducible nitric oxide synthase, as well as activation of nuclear factor-κB p65 in chondrocytes [22].

Doxycycline-induced apoptosis of H9c2 cells was studied to be protected and doxycycline-induced dissipation of Δ*Ψm* was recovered by 10 μM leonurine [23]. 10-Methoxy-leonurine (**1**) was first isolated from *L*. *japonicas* and reported to inhibit soluble epoxide hydrolases [17]. In this study, compounds **1** and **2** were identified as potential tyrosinase inhibitors that block the catalytic reactions of tyrosinase at approximately 10 μM. Upon binding to tyrosinase, both inhibitors blocked the interaction of the substrate with the tyrosinase active site. Molecular simulation is one of the essential methods for drug development by providing a clue to the ligand-receptor interaction by theoretical computational chemistry [24,25]. According to the molecular docking, the guanidine group occupied the cavity by three loops (Asn243 to er254, Gly62 to Leu89, and Arg321 to Gly330). Keton of ester in (**1**) was tied within 3.5 Å by His244, and the aromatic ring was anchored within the inner active site via hydrophobic interactions only. As a result, inhibitor (**1**) occupied a stable position extending from the cavity to the active site, whereas the hydroxyl group of the aromatic ring in (**2**) formed hydrogen bonds with His85, Asn81, Cys83, and Ala323, according to the top 10 ranks. The guanidine group of (**2**) was hung on the cavity of three loops, and the aromatic ring docked within the active site or the other. These findings likely explain the greater inhibitory activity of 10-methoxy-leonurine (**1**) than leonurine (**2**). Further studies will be necessary to increase the hydrophobicity of the aromatic ring of guanidine pseudoalkaloids for the development of novel tyrosinase inhibitors.

## 5. Conclusion

To identify novel tyrosinase inhibitors from plants, we prepared pure components from *L. japonicas*. Five compounds (**1**–**5**) were tested for their inhibitory activity against tyrosinase. The guanidine pseudoalkaloids 10-methoxy-leonurine (**1**) and leonurine (**2**) exhibited inhibitory activity against tyrosinase, with IC_50_ values of 7.4 ± 0.4 and 12.4 ± 0.8 μM, respectively. Based on enzyme kinetics and molecular docking, both compounds appear to operate as competitive inhibitors by locking the active site of the enzyme. In conclusion, compound (**1**), with a *K*_i_ value of 1.6 ± 0.7 μM, is a potential tyrosinase inhibitor, and guanidine pseudoalkaloids may be useful as lead compounds for the development of new tyrosinase agents.

## Figures and Tables

**Table 1:** Inhibitory activity of isolated compounds **1**–**5** from *L. japonicas* toward tyrosinase.

Inhibitor	Inhibition Rate (%) at 100 μM ^a^	IC_50_ Value (μM)	Binding Type (*K*_i_, μM)
**1**	91.8 ± 2.9	7.4 ± 0.4	Competitive (1.6 ± 0.7)
**2**	85.6 ± 1.8	12.4 ± 0.8	Competitive (11.4 ± 1.1)
**3**	11.6 ± 0.1	N.T ^c^	N.T ^c^
**4**	1.8 ± 5.9	N.T ^c^	N.T ^c^
**5**	8.3 ± 0.6	N.T ^c^	N.T ^c^
Kojic acid ^b^	79.4 ± 2.5	25.7 ± 1.3	

^a^ All compounds examined in a set of triplicated experiment; ^b^ Positive control; ^c^ Not tested.

**Table 2:** Autodock scores and hydrogen bonds of the top 10 ranks between tyrosinase and inhibitors.

Ranks	1		2	
	Autodock Score (kcal/mol)	Hydrogen Bonds (Å)	Autodock Score (kcal/mol)	Hydrogen Bonds (Å)
**1**	−6.83	His85 (2.69), His244(2.68), Gly245(3.05)	−6.16	Glu322(2.78)
**2**	−6.82	His244(2.99), Gly245(2.71)	−6.12	Ala246(2.67)
**3**	−6.78	His244(2.81), Gly245(2.97)	−6.12	His244(3.01), Ala246(2.67)
**4**	−6.76	His244(2.91), Gly245(2.65)	−6.1	Tyr78(3.07), Asn81(2.98), His85(2.50, 2.82)
**5**	−6.60	His244(2.95), Gly245(2.72)	−5.99	Asn81(2.64, 2.92), His85(2.67, 3.26), His244(3.02)
**6**	−6.58	Gly245(2.93)	−5.91	Asn81(2.84), Cys83(2.67) His85(3.01), Gly86(2.88),
**7**	−6.54	His85(2.82), His244(2.99), Glu322(2.56)	−5.85	His85(3.01), Gly86(3.04), Ala323(3.18)
**8**	−6.52	His244(2.87), Ala246(3.16)	−5.83	His85(3.03), Asn320(2.96)
**9**	−6.40	His85(3.05), His244(3.14), Asn320(3.01)	−5.80	His244(2.96), Glu322(2.68)
**10**	−6.38	His244(2.69), Gly245(3.08), Asn260(3.25)	−5.80	Asn81(2.63), Cys83(3.14), His85(2.85)

## Supplementary Materials

The following are available online at https://www.mdpi.com/2218-273X/10/2/174/s1, Figure S1–S10: ^1^H-NMR, ^13^C-NMR spectrum ESI-MS spectrum of compound **1**–**5**.

## References

[1] Fan M., Ding H., Zhang G., Hu X., Gong D. (2019). Relationships of dietary flavonoid structure with its tyrosinase inhibitory activity and affinity. LWT-Food Sci. Technol..

[2] Gui L., Lee J.h., Hao H., Park Y.-D., Zhan Y., Lü Z.-R. (2017). The effect of oxaloacetic acid on tyrosinase activity and structure: Integration of inhibition kinetics with docking simulation. Int. J. Biol. Macromol..

[3] Carcelli M., Rogolino D., Bartoli J., Pala N., Compari C., Ronda N., Bacciottini F., Incerti M., Fisicaro E. (2020). Hydroxyphenyl thiosemicarbazones as inhibitors of mushroom tyrosinase and antibrowning agents. Food Chem..

[4] Zeng H.-J., Liu Z., Hu G.-Z., Qu L.-B., Yang R. (2019). Investigation on the binding of aloe-emodin with tyrosinase by spectral analysis and molecular docking. Spectrochim. Acta. A Mol. Biomol. Spectrosc..

[5] Dehghani Z., Khoshneviszadeh M., Khoshneviszadeh M., Ranjbar S. (2019). Veratric acid derivatives containing benzylidene-hydrazine moieties as promising tyrosinase inhibitors and free radical scavengers. Bioorg. Med. Chem. Lett..

[6] Cásedas G., Les F., González-Burgos E., Gómez-Serranillos M.P., Smith C., López V. (2019). Cyanidin-3-*O*-glucoside inhibits different enzymes involved in central nervous system pathologies and type-2 diabetes. S. Afr. J. Bot..

[7] Tian J.-L., Liu T.-L., Xue J.-J., Hong W., Zhang Y., Zhang D.-X., Cui C.-C., Liu M.-C., Liu S.-L. (2019). Flavanoids derivatives from the root bark of *Broussonetia papyrifera* as a tyrosinase inhibitor. Ind. Crop. Prod..

[8] Wagle D., Seong S.H., Jung H.A., Choi J.S. (2019). Identifying an isoflavone from the root of *Pueraria lobata* as a potent tyrosinase inhibitor. Food Chem..

[9] Tan Y.-J., Xu D.-Q., Yue S.-J., Tang Y.-P., Guo S., Yan H., Zhang J., Zhu Z.-H., Shi X.-Q., Chen Y.-Y. (2020). Comparative analysis of the main active constituents from different parts of *Leonurus japonicus* Houtt. and from different regions in China by ultra-high performance liquid chromatography with triple quadrupole tandem mass spectrometry. J. Pharm. Biomed. Anal..

[10] Zhong W.-M., Cui Z.-M., Liu Z.-K., Yang Y., Wu D.-R., Liu S.-H., Long H., Sun H.-D., Dang Y.-J., Xiao W.-L. (2015). Three minor new compounds from the aerial parts of *Leonurus japonicas*. Chinese Chem. Lett..

[11] Li H.-Y., Peng X., Jin X.J., Wei W.-J., Ma K.-L., Li Y., Chen J.-J., Gao K. (2019). Labadane-type diterpenoids from *Leonurus japonicus* and their plant-growth regulatory activity. J. Nat. Prod..

[12] Li Y., Chen Z., Feng Z., Yang Y., Jiang J.S., Zhang P.C. (2012). Hepatoprotective glycosides from *Leonurus japonicas* Houtt. Carbohydr. Res..

[13] Zhou Q.-m., Peng C., Yang H., Liu L.-S., Yang Y.-T., Xie X.-F., Guo L., Liu Z.-H., Xiong L. (2015). Steroids from the aerial parts of *Leonurus japonicus*. Phytochem. Lett..

[14] Liu J., Peng C., Zhou Q.-M., Guo L., Liu Z.-H., Xiong L. (2018). Alkaloids and flavonoid glycosides from the aerial parts of *Leonurus japonicas* and their opposite effects on uterine smooth muscle. Phytochemistry.

[15] Lai K.-Y., Hu H.-C., Chiang H.-M., Liu Y.-J., Yang J.-C., Lin Y.-A., Chen C.-J., Chang Y.-S., Lee C.-L. (2018). New diterpenes leojaponins G-L from *Leonurus japonicas*. Fitoterapia.

[16] Santi M.D., Bouzidi C., Gorod N.S., Puiatti M., Michel S., Grougnet R., Ortega M.G. (2019). In vitro biological evaluation and molecular docking studies of natural and semisynthetic flavones from *Gardenia oudiepe* (Rubiaceae) as tyrosinase inhibitors. Bioorg. Chem..

[17] Leem H.H., Lee G.Y., Lee J.S., Lee H.N., Kim J.H., Kim Y.H. (2017). Soluble epoxide hydrolase inhibitory activity of components from *Leonurus japonicas*. Int. J. Biol. Macromol..

[18] Wang L., Yang X., Qin P., Shan F., Ren G. (2013). Flavonoid composition, antibacterial and antioxidant properties of tartary buckwheat bran extract. Ind. Crops. Prod..

[19] Ghafary S., Ranjbar S., Larijani B., Amini M., Biglar M., Mahdavi M., Bakhshaei M., Khoshneviszadeh M., Sakhteman A., Khoshneviszadeh M. (2019). Novel morpholine containing cinnamoyl amides as potent tyrosinase inhibitors. Int. J. Biol. Macromol..

[20] D’Mello S.A.N., Finlay G.J., Baguley B.C., Askarian-Amiri M.E. (2016). Signaling pathways in melanogenesis. Int. J. Mol. Sci..

[21] Kong Y.C., Yeung H.W., Cheung Y.M., Phil M., Hwang J.C., Chan Y.W., Law Y.P., Ng K.H., Yeung C.H. (1976). Isolation of the uterotonic principle from *Leonurus artemisia*, the Chinese Motherwork. Am. J. Chin. Med..

[22] Yin W., Lei Y. (2018). Leonurine inhibits IL-1β induced inflammation in murine chondrocytes and ameliorates murine osteoarthritis. Int. Immunopharmacol..

[23] Xin H., Liu X.H., Zhu Y.Z. (2009). *Herba leonurine* attenuates doxorubicin-induced apoptosis in H9c2 cardiacmuscle cell. Eur. J. Pharmacol..

[24] Pradeepkiran J.A., Reddy P.H. (2019). Structure based design and molecular docking studies for phosphorylated tau inhibitors in Alzheimer’s disease. Cells.

[25] Pradeepkiran J.A., Reddy A.P., Reddy P.H. (2019). Pharmacophore-based models fro therapeutic drugs against phosphorylated tau in Alzheimer’s disease. Drug Discov. Today.

