# Supplementary material for: The Guanidine Pseudoalkaloids 10-Methoxy-Leonurine and Leonurine Act as Competitive Inhibitors of Tyrosinase
Source: Biomolecules. 2020 Jan 23;10(2):174. doi: 10.3390/biom10020174 (PMC7072302; doi:10.3390/biom10020174)
Supplement: Supplementary file 1 [file biomolecules-10-00174-s001.pdf]

The guanidine pseudoalkaloids 10-methoxy-leonurine and leonurine acts as competitive inhibitors of tyrosinase

Jang Hoon Kim[a,]**, Hyun Hee Leem[b], Ga Young Lee[c,]**

[a]Research Institute for Basic Sciences, JeJu National University, Jeju 63243, Republic of Korea
[b]National Development Institute of Korean Medicine, Gyeongsan 38573, Republic of Korea
[c]Hephzibah Korea Lnc, Techno 10-ro, Yuseong-gu, Daejeon 34036, Republic of Korea

****Corresponding authors**

Tel.:+82-64-754-8308; E-mail address:oasis5325@gmail.com (J.H. Kim).

Tel.:+82-42-935-9944; Fax: +82-42-935-9955; E-mail address:gylee6409@gmail.com (G.Y. Lee).

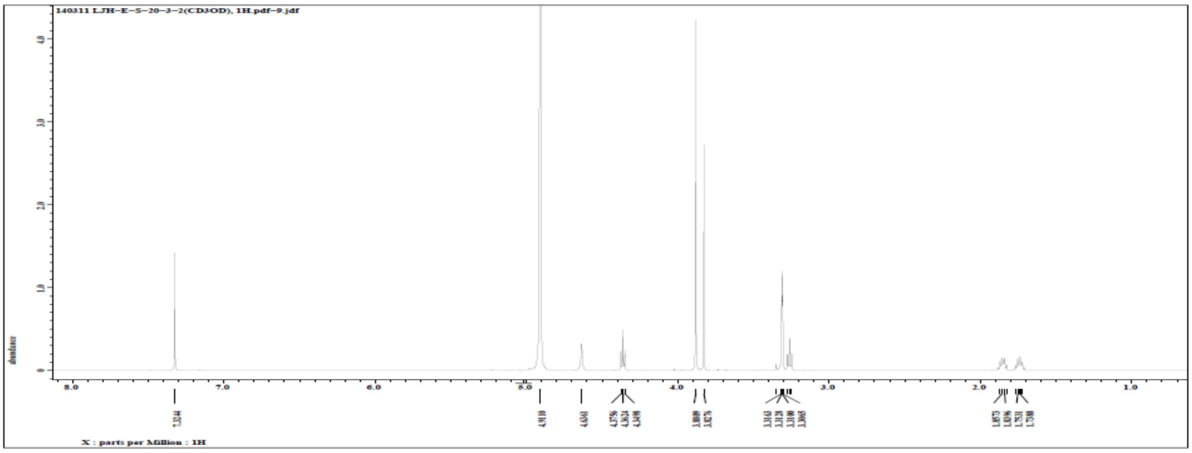

Figure S1.¹H NMR spectrum of compound 1. (methanol-d_4, 400 MHz)

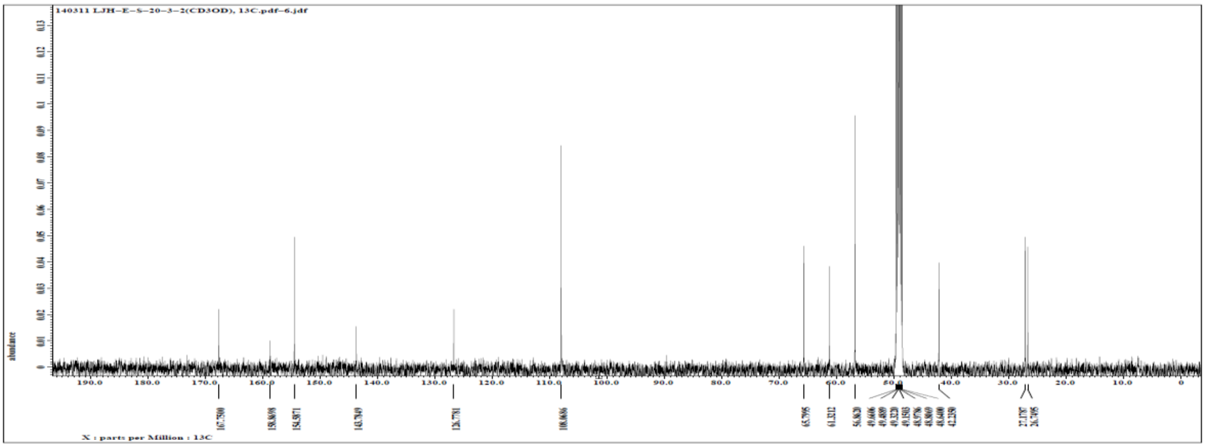

Figure S2.¹³C NMR spectrum of compound 1. (methanol-d_4, 100 MHz)

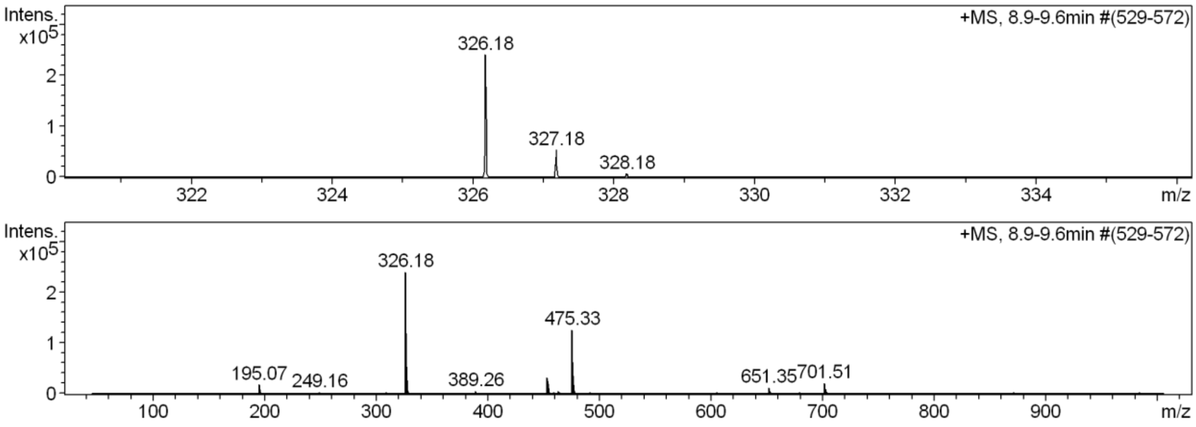

Figure S3.ESI-MS spectrum of compound 1.

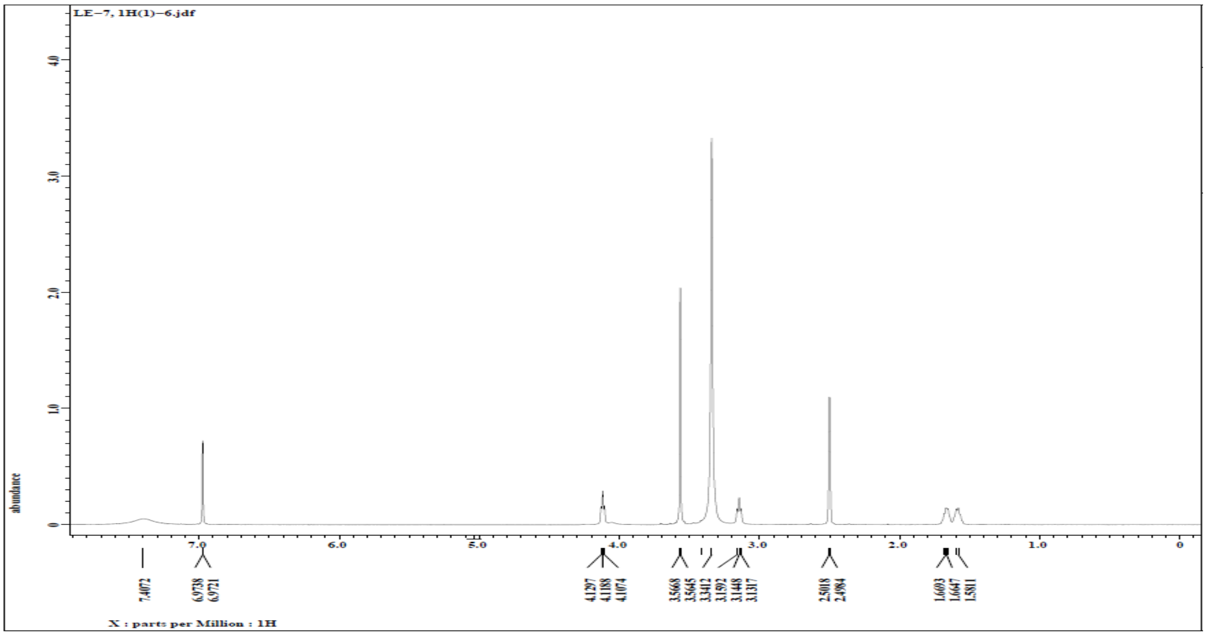

Figure S4. **^{1}H NMR spectrum of compound 2 (DMSO-d_6, 300 MHz).**

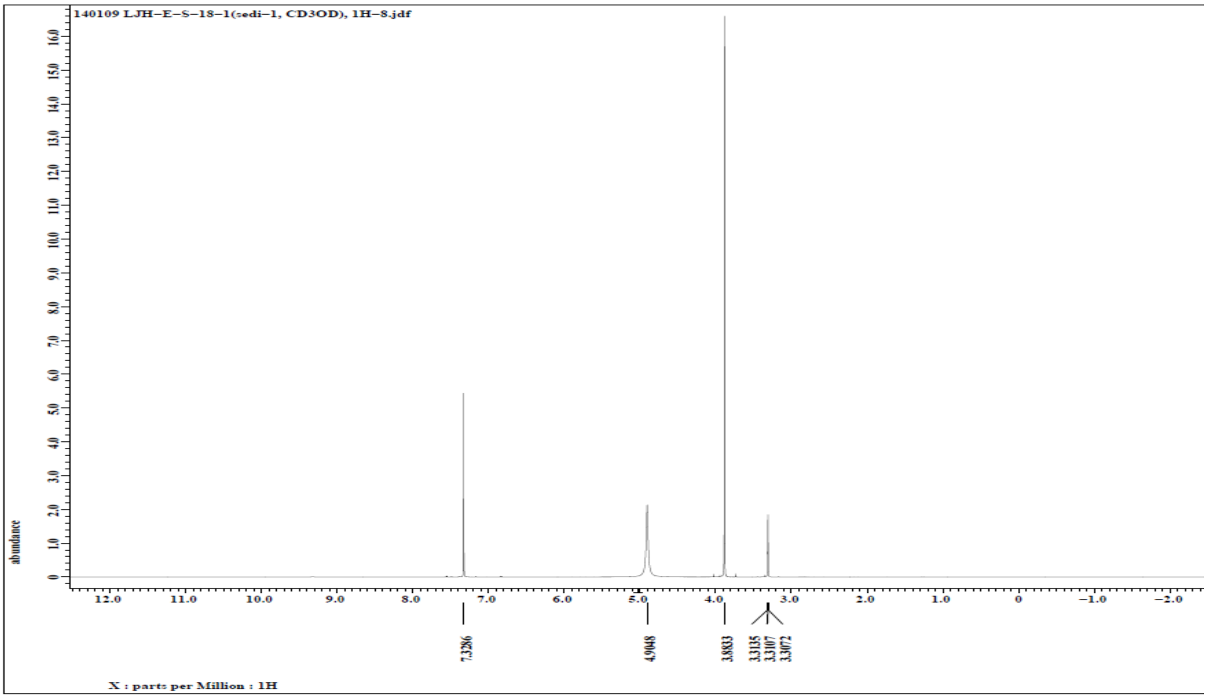

Figure S5. ^{1}**H NMR spectrum of compound 3 (**CD_3OD**, 500 MHz).**

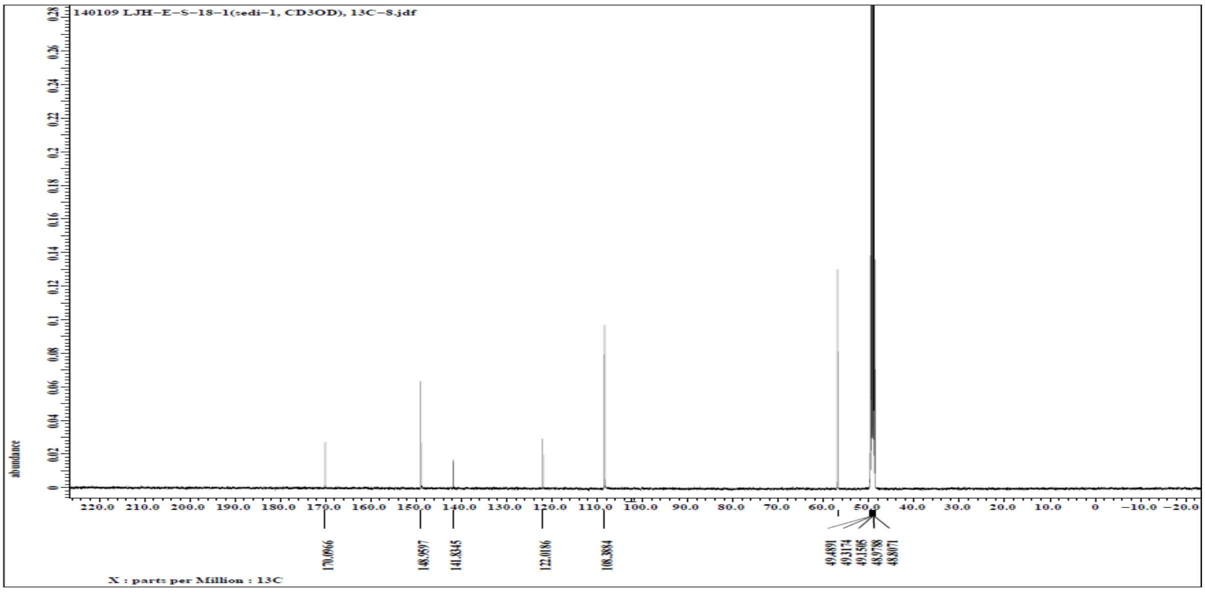

Figure S6. ^{13}**C NMR spectrum of compound 3 (**CD_3OD**, 125 MHz).**

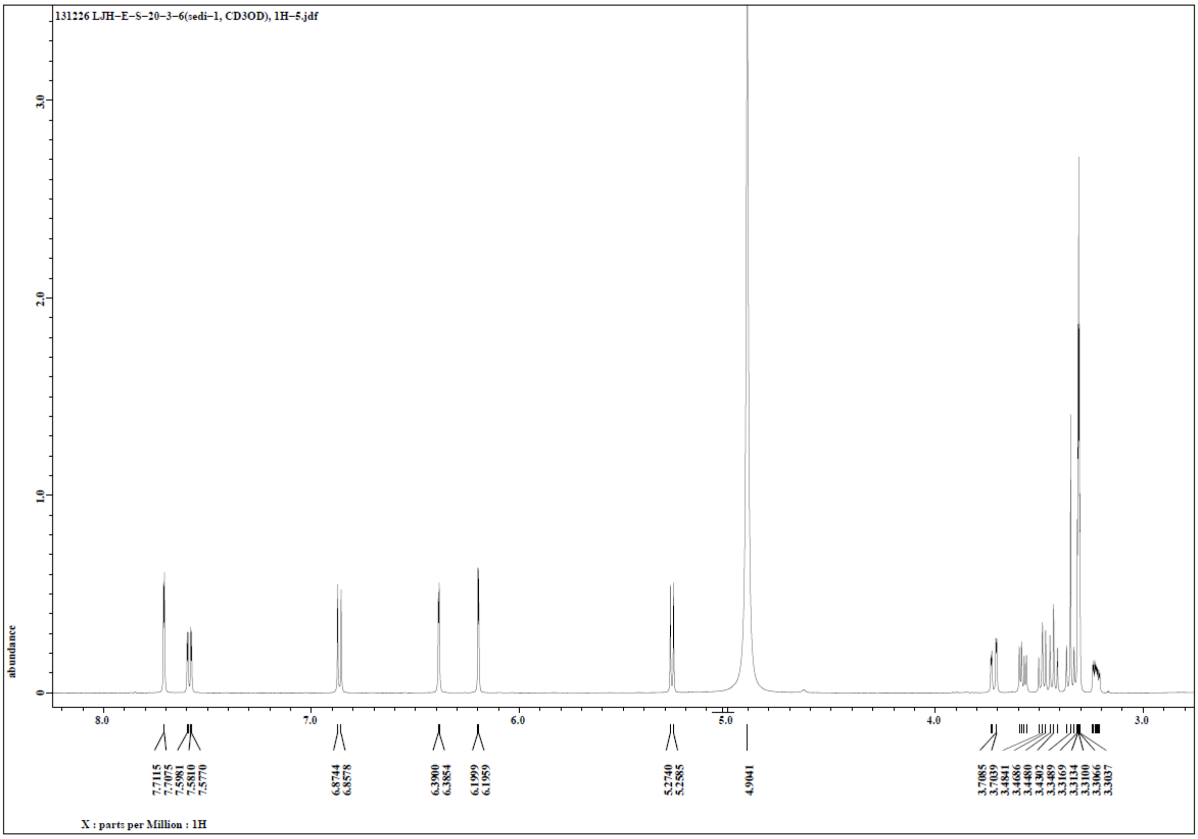

Figure S7.^{1}H NMR spectrum of compound 4 (CD_3OD, 500 MHz).

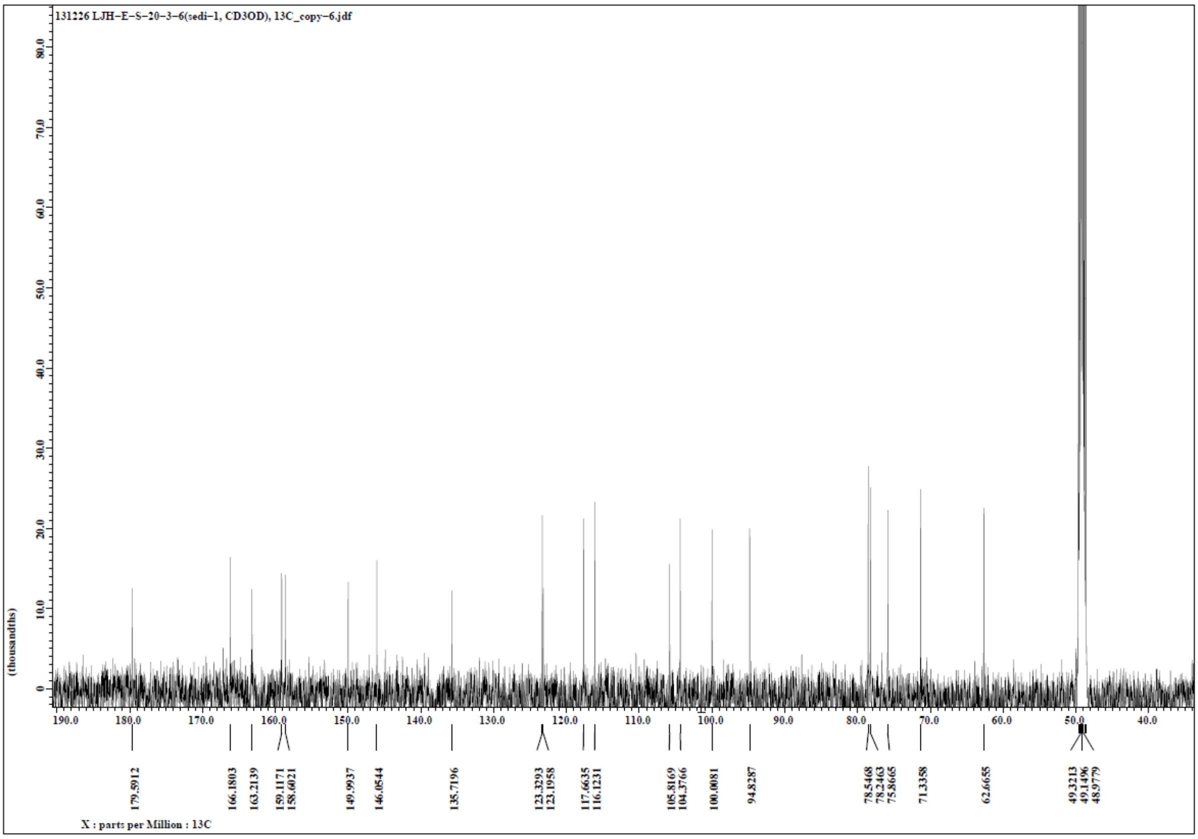

Figure S8.^{13}C NMR spectrum of compound 4 (CD_3OD, 125 MHz).

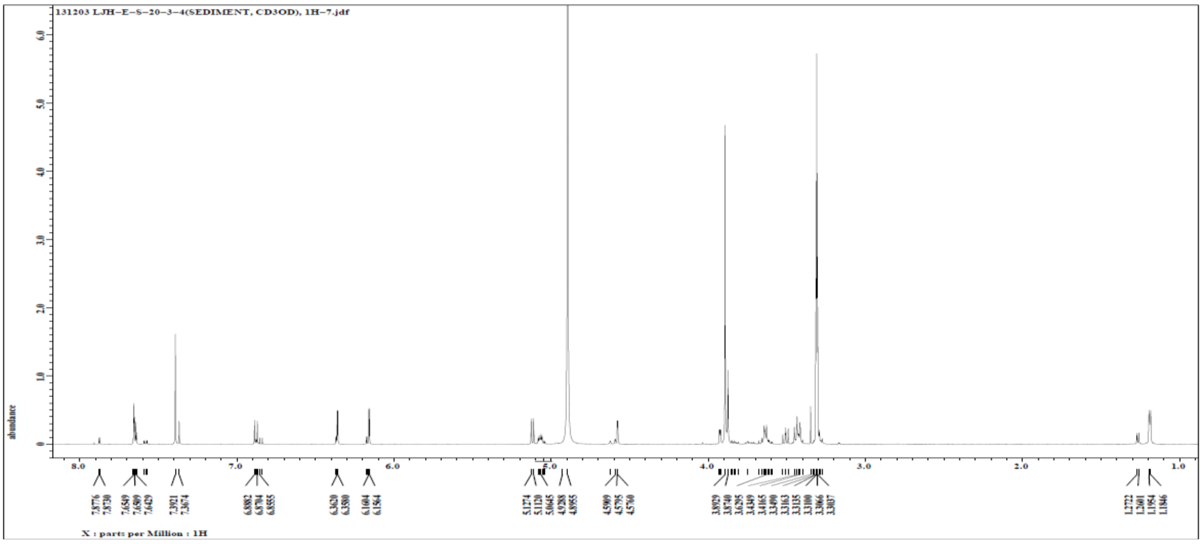

Figure S9.^{1}H NMR spectrum of compound 5 (CD_3OD, 500 MHz).

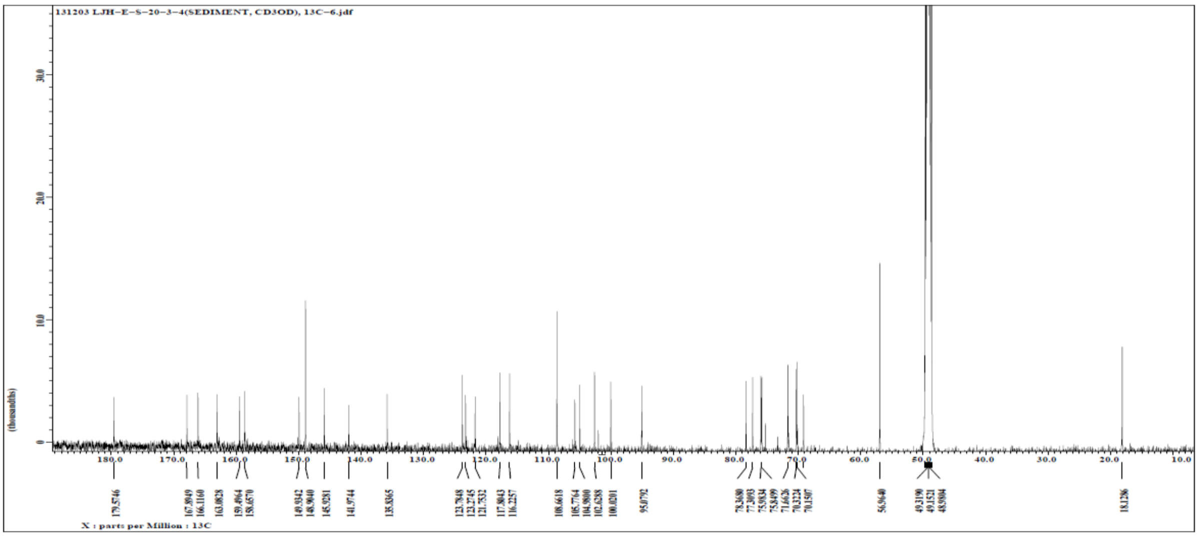

Figure S10.^{13}C NMR spectrum of compound 5 (CD_3OD, 125 MHz).
